# Compensatory responses of the insulin signaling pathway restore muscle glucose uptake following long-term denervation

**DOI:** 10.14814/phy2.12359

**Published:** 2015-04-20

**Authors:** Zachary J Callahan, Michael Oxendine, Joshua L Wheatley, Chelsea Menke, Emily A Cassell, Amanda Bartos, Paige C Geiger, Paul J Schaeffer

**Affiliations:** 1 Department of Biology, Miami UniversityOxford, Ohio; 2 Department of Molecular and Integrative Physiology, University of Kansas Medical CenterKansas City, Kansas

**Keywords:** Akt, diabetes, disuse atrophy, nerve transection

## Abstract

We investigated the role of muscle activity in maintaining normal glucose homeostasis via transection of the sciatic nerve, an extreme model of disuse atrophy. Mice were killed 3, 10, 28, or 56 days after transection or sham surgery. There was no difference in muscle weight between sham and transected limbs at 3 days post surgery, but it was significantly lower following transection at the other three time points. Transected muscle weight stabilized by 28 days post surgery with no further loss. Myocellular cross-sectional area was significantly smaller at 10, 28, and 56 days post transection surgery. Additionally, muscle fibrosis area was significantly greater at 56 days post transection. In transected muscle there was reduced expression of genes encoding transcriptional regulators of metabolism (PPAR*α*, PGC-1*α*, PGC-1*β*, PPAR*δ*), a glycolytic enzyme (PFK), a fatty acid transporter (M-CPT 1), and an enzyme of mitochondrial oxidation (CS) with transection. In denervated muscle, glucose uptake was significantly lower at 3 days but was greater at 56 days under basal and insulin-stimulated conditions. Although GLUT 4 mRNA was significantly lower at all time points in transected muscle, Western blot analysis showed greater expression of GLUT4 at 28 and 56 days post surgery. GLUT1 mRNA was unchanged; however, GLUT1 protein expression was also greater in transected muscles. Surgery led to significantly higher protein expression for Akt2 as well as higher phosphorylation of Akt. While denervation may initially lead to reduced glucose sensitivity, compensatory responses of insulin signaling appeared to restore and improve glucose uptake in long-term-transected muscle.

## Introduction

Incidence of type 2 diabetes is rising worldwide and the number of affected individuals is growing much faster than anticipated. In 1997 it was estimated that by 2020 the number of type 2 diabetics would be approximately 250 million people worldwide (Miura et al. 2003). However, two recent studies show that figure has already been eclipsed with an estimated number of adults with diabetes ranging from 287 million in 2009 (Salonen et al. 1985) to 347 million in 2011 (Danaei et al. 2011). Calculations using the lower value yielded a prediction of 439 million affected individuals by 2030 (Salonen et al. 1985), with huge increases (up to 98% in some countries) seen throughout the world.

Clearly, many factors play a role in the pathogenesis of diabetes, but one of the primary components is activity. The benefits of increased activity in preventing metabolic diseases, including type 2 diabetes, have been discussed previously and include large reductions (58%) in the risk of impaired glucose tolerance progressing to type 2 diabetes, decreases in mortality, as well as improvements in blood pressure and blood lipids (Shulman 2000). Even brief light-to-moderate activity has a beneficial impact on the expression of genes involved in metabolism, as well as improving glucose metabolism (Kramer et al. 2006). However, the effects of disuse atrophy on the regulation of normal metabolic function are less understood. As obesity is often associated with disuse atrophy, research isolating the effects of disuse atrophy on glucose uptake is ongoing. Attempts to mimic the effects of disuse atrophy have included suspension of the animal by the tail or immobilizing one hindlimb via denervation or stapling. Denervation has frequently been used to disrupt activity on a local scale and has been shown to impair glucose uptake as soon as 24 h after surgery (Thomason et al. 1987; Block et al. 1991; Henriksen et al. 1991; Lin et al. 2004). However, denervation of skeletal muscle initiates a cascade of events including rapid upregulation of factors involved in apoptotic and necrotic pathways which further compromise normal cellular function, potentially including glucose uptake, as well as causing significant decreases in muscle mass (Thomason et al. 1987; Henriksen et al. 1991; Lin et al. 2004). As previous studies have focused on short-term effects of denervation, it remains unclear whether impaired glucose uptake is due to disuse atrophy, active apoptosis and necrosis, or a combination of these effects. It is well established that severe reductions in skeletal muscle mass due to denervation are temporally limited to the first 25–30 days, after which time the reduced mass is maintained indefinitely (Talanian et al. 2010). By extending our investigation beyond the time frame when apoptosis and necrosis are highly active, we will better elucidate the ability of skeletal muscle to import glucose during prolonged disuse atrophy.

Plasma glucose is imported intra-cellularly via the family of glucose transporters, including GLUT 1 and GLUT 4 (Wagatsuma 2006). GLUT1 is distributed throughout the plasma membrane and functions in basal glucose uptake. GLUT4, however, is the primary transport mechanism for insulin-stimulated glucose uptake in both skeletal muscle and adipose tissue (Shaw et al. 2010). An increase in insulin binding to its receptor initiates a signaling cascade that ultimately results in the translocation of GLUT4 from vesicles inside the cell to the cell membrane allowing facilitated diffusion of glucose into the cell (O'Rahilly 1997; Kamei et al. 2003). It has been shown that exercise improves the capacity of skeletal muscle to import glucose into the cell (Ivy et al. 1983). It has been proposed that disuse atrophy alone could promote pathogenesis of diabetes and other metabolic diseases (Lee et al. 2006), but the effects disuse atrophy has on glucose uptake or GLUT expression are less understood.

Here, we examine for the first time the effect of long-term denervation on glucose uptake in skeletal muscle. In addition, we investigate expression of genes involved in mitochondrial oxidation, substrate transport, and regulation of metabolism. For these parallel processes, we have two hypotheses: (1) as demonstrated previously, transection surgery will lower glucose uptake in the days immediately following surgery, but glucose uptake will be improved in the later time points; and (2) transection surgery will initially lower expression of genes involved in regulation of metabolism, as well as substrate uptake and oxidation. We expect expression of these genes to improve with time as well.

## Materials and Methods

### Animals and surgery

All animal experiments and euthanasia protocols were conducted in accordance with the National Institutes of Health guidelines for humane treatment of laboratory animals and were reviewed and approved by the Institutional Animal Care and Use Committee of Miami University.

C57BL/6J mice were obtained from Harlan Laboratories (Indianapolis, IN) and a breeding colony was established at the Miami University Animal Care Facility (except those used for the functional glucose uptake). All mice were housed in cages under conditions of controlled temperature and humidity with a 12:12-h light:dark cycle and given standard chow and water ad libitum. Upon reaching 8 weeks of age, mice were anesthetized with isofluorane delivered via inhalation at 5% for induction, followed by reduction to 2% for maintenance. One hindlimb was denervated by surgical removal of a 1.0 cm segment of the sciatic nerve, which innervates the tibialis anterior (TA), extensor digitorum longus (EDL), gastrocnemius (GC), and soleus (SOL) muscles, as previously described (Thomason and Booth 1990). On the contralateral (sham) hindlimb, the sciatic nerve was visualized but not touched. After surgery, the mice were randomly assigned for sacrifice at 3, 10, 28, or 56 days post surgery. Insulin (0.5U/mouse) or saline was injected (intraperitoneal) 10 min prior to sacrifice. Mice were killed by carbon dioxide inhalation, weighed and the hindlimb muscles were removed, weighed and frozen for subsequent analysis, except for the mice used for the glucose uptake assays (see below). In each of the experimental time points, muscles from the contralateral limb were used as an internal control.

C57BL/6J mice used in the glucose uptake assay were also obtained from Harlan Laboratories but were housed at the Kansas University Medical Center. Mice were housed and denervation surgery performed as above. After surgery, the mice were randomly assigned to glucose uptake experiments at 3, 10, 28, or 56 days post surgery. Upon reaching terminal end points, pentobarbital was used to anesthetize the mice. Prior to euthanasia EDL muscles were removed, weighed and uptake experiments performed as described below.

### Histological analysis

Tibialis anterior muscles from control and denervated legs were frozen in liquid nitrogen-cooled 2-methyl butane and stored at −80°C. Cross sections (12 *μ*m) were cut using a cryostat (Micron HM 550; Thermo Scientific, Florence, KY) and sections were stained with Gomori trichrome following Engel and Cunningham (1963). ImageJ software (NIH) was used to measure total area of each section, area containing fibrotic tissue, and myocellular cross-sectional area.

### Protein analysis

Snap frozen GC samples were sonicated in RIPA buffer. Total protein concentration in muscle homogenates was determined via BCA assay. 50 *μ*g of muscle protein extracts from GC was resolved by SDS-PAGE (7.5%) and subsequently transferred to nitrocellulose membranes. Membranes were blocked (1 h at room temperature) with a 5% skim milk (5% BSA was used for p-Akt) in TBS-T [Tris-buffered saline and 0.1% Tween 20] solution. Blots were then incubated overnight at 4°C with anti-rabbit antibody directed against GLUT4 (ab654) or GLUT1 (ab652) (Abcam, Cambridge, MA), Akt2 (3063) or phospho-Akt Ser473 (9271) (Cell Signaling, Beverly, MA). After primary antibody incubation, blots were washed four times in TBS-T (1 × 15, 3 × 5 min.) and incubated with horseradish peroxidase (HRP)-linked goat anti-rabbit secondary antibody (Cell Signaling Technology; 1:1000 dilution) at room temperature (60 min). Protein expression was assayed using enhanced chemiluminescence (Pierce-Thermo Fisher Scientific, Rockford, IL.) and quantified using Alphamager HP (Protein Simple, San Jose, CA). Values for 3-day sham were used to determine relative expression and Ponceau S staining was used to verify loading volumes. Negative and positive loading controls for GLUT4, GLUT1, Akt2, and p-Akt were not used.

### Quantitative RT-PCR

Quantitative RT-PCR using 36B4 (acidic ribosomal phosphoprotein P0) as a control gene was used to assess expression levels of transcripts for various genes including: GLUT1 (Glucose transporter type 1), GLUT4 (Glucose transporter type 4), PPAR*α* and PPAR*δ* (Peroxisome proliferator-activated receptor alpha and beta/delta), PGC-1*α* and PGC-1*β* (PPAR gamma coactivator 1-alpha and beta), CD36 (Fatty Acid Translocase), MCAD (Medium-chain acyl-CoA dehydrogenase), PFK (Phosphofructokinase), M-CPT I (Muscle Carnitine Palmitoyltransferase I) and CS (Citrate Synthase). Primer efficiencies were verified for all genes investigated and all gene expression data were normalized to 36B4, which did not change with treatment.

Primers were obtained from Integrated DNA Technologies (Corralville, IA); see Table S1 for primer sequences. RNA was isolated from GC muscle using TRIzol (Life Technologies, Grand Island, NY) according to manufacturer's instructions. RNA quality was assessed spectrophotometrically (NanoDrop 1000, NanoDrop-Fisher Thermo Scientific, Rockford, IL) and quantified by absorption spectrophotometry at 260 and 280 nm. cDNA was generated from the total RNA for each muscle using qScript™ cDNA Synthesis Kit (Quanta Biosciences, Gaithersburg, MD) according to the manufacturer's instructions. Relative quantitative RT-PCR was subsequently performed with a RotorGene 3000 (Qiagen, Valencia, CA) system using SYBR Green Master Mix (Applied Biosystems, Foster City, CA). For each gene, real-time PCR was performed in triplicate wells on cDNA generated from the reverse transcription of 10 ng of total RNA.

### Functional glucose uptake

Freshly harvested EDL muscles were placed in Recovery buffer for 60 min at 35°C, then rinsed for 10 min at 29°C in 2 mL of oxygenated Krebs Henseleit Buffer (KHB) containing 40 mmol/L mannitol to remove glucose from the extracellular space. After the rinse step, muscles were incubated for 20 min at 29°C in flasks containing 2 mL of KHB with 1 mmol/L 2-deoxy-[1,2-3H] glucose (2-DG; 1.5 *μ*Ci/mL) and 36 mmol/L [14C] mannitol (0.2 *μ*Ci/mL), with a gas phase of 95% O_2_–5% CO_2_, in a shaking incubator. The same additions that were in the rinse were present during the determination of glucose transport. The muscles were then blotted and clamp frozen and processed for determination of intracellular 2-DG accumulation and extracellular space, as described previously (Geiger et al. 2006; Wofford et al. 2008). 2-Deoxy-[1,2-3H] glucose was purchased from American Radiolabeled Chemicals (St. Louis, MO). [14C] mannitol was obtained from ICN Radiochemicals (Irvine, CA).

### Statistical analysis

Tissue morphology, quantitative PCR, and Western blot expression data as well as glucose uptake were analyzed using an analysis of variance (ANOVA) with surgery and time (and insulin for the glucose uptake assay) as factors using JMP statistical software (version 10.0.0). When significant differences were detected in parameters, pairwise comparisons were run using the Tukey HSD method. Pairwise comparisons are reported in the running text of the results section. The level of significance was set at *P *< 0.05.

Data are presented as means ± SEM (*n*).

## Results

### Tissue weight and morphology

**Figure 1:**
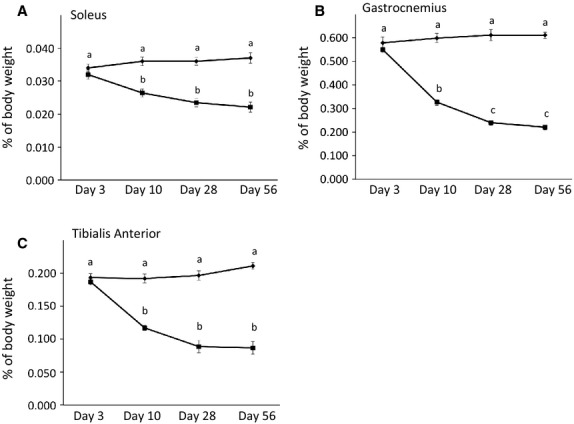
Denervation leads to rapid initial weight loss in hind-limb muscles with no subsequent loss after 28 days post surgery. A. Soleus B. Gastrocnemius C. Tibialis Anterior. Muscle weight is expressed as a percentage of body weight (n = 18 for each). Values are means ± SEM. Different superscripted letters indicate significant pairwise differences between groups following significant time by surgery interaction (ANOVA *P *< 0.05.

There was a significant effect of time and surgery on weight of GC and TA as well as a significant effect of surgery on SOL muscles (ANOVA, see Table1 for all ANOVA *P*-values). The interaction between surgery and time was also significant for all three muscles (*P *< 0.05). There was not a significant difference in weight for any of the three muscles taken from the sham surgery limbs at any time point. Pairwise comparison revealed that transection surgery did not cause differences in muscle weight at 3 days post surgery. In 10 days, however, the muscle weight (as a percentage of body weight) of the SOL, GC, and TA was significantly lower in the transected limbs as compared to sham surgery (*P *< 0.05; Fig.1). Weight of the GC was lower still at 28 days, and did not change thereafter, while weight remained constant for the SOL and TA after day 10 (*P *< 0.05; Fig.1).

**Table 1:** Outcome of analysis of variance ANOVA (*P*-values) for all variables except glucose uptake.

Variable	Time	Surgery	Interaction
Soleus weight	0.0916	<0.0001	<0.0001
Gastrocnemius weight	<0.0001	<0.0001	<0.0001
Tibialis anterior weight	<0.0001	<0.0001	<0.0001
% Fibrosis	0.0007	0.0001	0.0987
Mean CSA	0.0287	<0.0001	0.1912
PPAR*α* mRNA	0.8495	<0.0001	0.5041
PPAR*δ* mRNA	0.0006	<0.0001	0.5508
PGC-1*α* mRNA	0.4102	<0.0001	0.9486
PGC-1*β* mRNA	0.0293	<0.0001	0.0217
MCPT-1 mRNA	0.0561	<0.0001	0.7904
MCAD mRNA	0.4878	<0.0001	0.7829
CD36 mRNA	0.9574	<0.0001	0.9405
PFK mRNA	0.5778	<0.0006	0.2899
CS mRNA	0.9991	<0.0001	0.2829
GLUT4 mRNA	0.4939	<0.0001	0.9667
GLUT1 mRNA	0.4739	0.2010	0.6312
GLUT4 Protein	0.0016	0.0124	0.0019
GLUT1 Protein	0.3399	<0.0001	0.5571
Akt2 Protein	0.5685	<0.0001	0.9227
p-Akt Protein	0.1057	0.0014	0.7262

**Figure 2:**
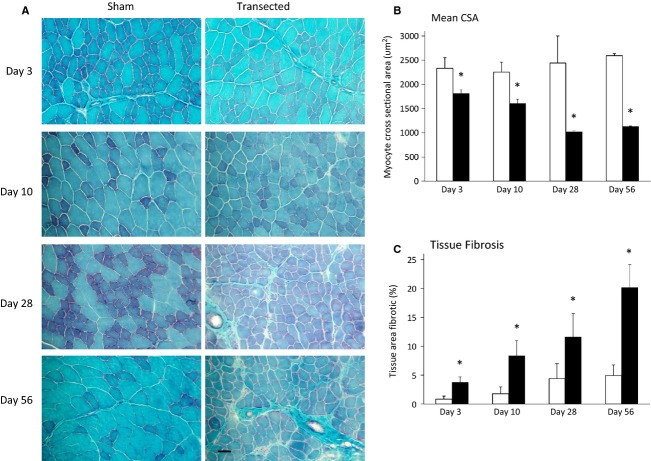
Myocellular cross-sectional area is reduced following denervation surgery. A. Representative images of fiber cross sectional sections stained with Gomori-trichrome. B. Myocellular cross-sectional area is lower in cross section of TA muscle with time. (n = 5). C. Myocellular area exhibiting fibrosis is higher in denervation muscles. (n = 5). Open bars represent sham surgery while closed bars represent denervation surgery. Values are means ± SEM, (n = 9−11). *indicates a significant effect of surgery (ANOVA *P *< 0.05).

ANOVA revealed that time and surgery each had a significant effect on percent fibrotic area and myocyte cross-sectional area (CSA), (*P *< 0.05). Histological analysis via Gomori trichrome staining showed that the transected muscles had significantly greater fibrotic area (green coloration – indicating an increase in interstitial collagen) than sham surgery (*P *< 0.05; Fig.2A and C). Concordantly, the mean CSA was significantly lower (*P *< 0.05; Fig.2A and B) in the transected muscles.

### Expression of metabolic genes

**Figure 3:**
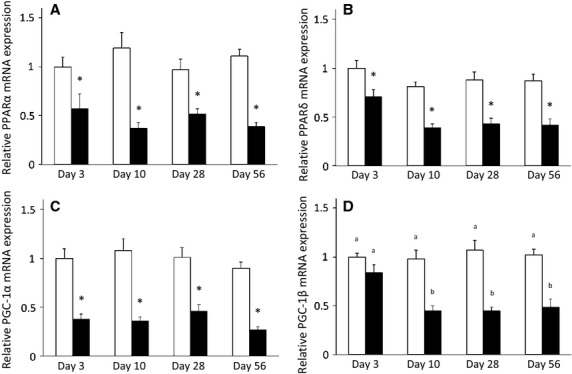
mRNA expression is lower for genes involved in metabolic regulation in denervated GC muscle throughout time course of study. A. PPAR B. PPAR*δ*; C. PGC-1*α* D. PGC-1*β*. Open bars represent sham surgery while closed bars represent denervation surgery. Values are means ± SEM, (n = 9−11). Quantitative data for each gene were normalized and corrected for the expression of a housekeeping gene (36B4). *indicates a significant effect of surgery (ANOVA *P *< 0.05). Different superscripted letters indicate significant pairwise differences between groups following significant time by surgery interaction (ANOVA *P *< 0.05).

**Figure 4:**
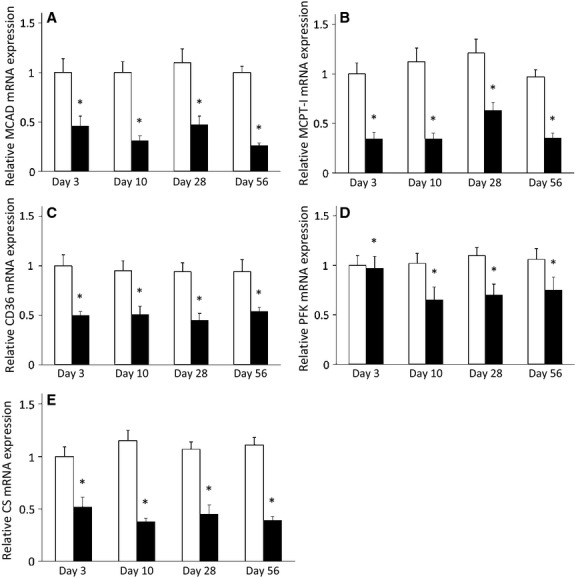
mRNA expression is lower for genes regulating metabolic enzymes in denervated GC muscle throughout time course of study. A. MCAD; B. MCPT-1; C. CD36; D. PFK; E. CS. Open bars represent sham surgery while closed bars represent denervation surgery. Values are means ± SEM, (n = 9–11). Quantitative data for each gene were normalized and corrected for the expression of a housekeeping gene (36B4). *indicates a significant effect of surgery (ANOVA *P *< 0.05).

We investigated genes that are involved in regulation of metabolic function and found that time had a significant effect on gene expression for PPAR*δ* and PGC-1*β*, with surgery having an effect on PPAR*α*, PPAR*δ*, PGC-1*α*, and PGC-1*β* (*P *< 0.05). The only significant interaction between time and surgery was for the expression of PGC-1*β* (*P *< 0.05). There was no significant difference in the expression levels for any of these genes at any time point in the sham surgery muscles (*P *> 0.05; Fig.3). PPAR*α*, PPAR*δ*, and PGC-1*α* expression after transection was significantly lower at all time points as compared to sham surgery (*P *< 0.05; Fig.3). Although later to develop, muscle from the transected limbs showed a significant decrease in the expression of PGC-1*β* at 10, 28, and 56 days post surgery (*P *< 0.05; Fig.3). Surgery also led to significantly lower expression levels of genes expressing metabolic enzymes (MCAD, MCPT1, CD36, PFK, and CS) in the transected muscles (*P *< 0.05) and none showed a significant interaction effect (*P *> 0.05); Fig.4).

### Functional glucose uptake and glucose transporter expression

**Figure 5:**
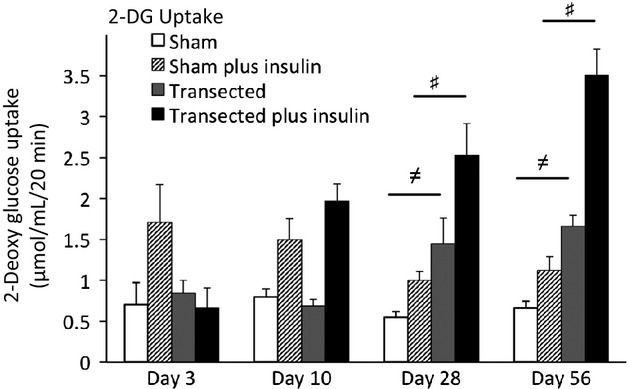
Skeletal muscle glucose uptake and transport improves in response to chronic denervation over the course of long-term denervation. 2-DG uptake is greater in chronically denervated EDL muscle. Sham (open bar), sham plus insulin (diagonal stripes), denervated (gray bar), denervated plus insulin (closed bar). Values are means ± SEM, (n = 7– 9), *P *< 0.05. Significant pairwise differences between transected and sham surgery groups after insulin treatment is indicated by #. Significant pairwise differences between transected and sham surgery groups before insulin treatment is indicated by ≠.

There was a significant effect of time, surgery and their interaction on glucose uptake (*P *< 0.05). The functional glucose uptake assay performed on the EDL muscle demonstrated a blunted response to insulin stimulation in the transected muscles at 3 days post surgery, but an increase in insulin response at 28 and 56 days post surgery (*P *< 0.05; Fig.5). Pairwise comparison showed that basal glucose uptake was significantly higher at 28 and 56 days post surgery (*P *< 0.05; Fig.5).

**Figure 6:**
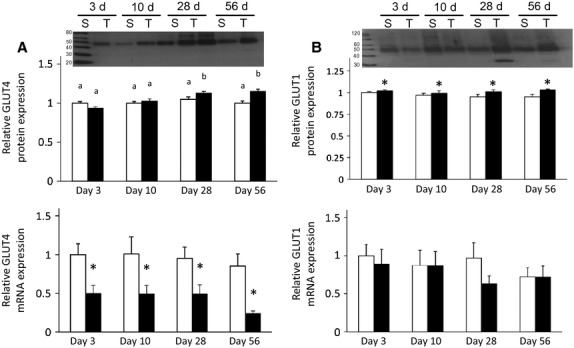
A. GLUT4 protein expression (top) is higher in chronically denervated GC muscle while GLUT4 gene expression (bottom) is lower in chronically denervated GC muscle. B. GLUT1 protein expression (top) is higher in chronically denervated GC muscle. GLUT1 gene expression (bottom) is not different in chronically denervated GC muscle. Expression presented relative to 3-day sham. Ponceau S staining was used to verify loading volumes for Western blots. Values are means ± SEM, (n = 7–9). *indicates a significant effect of surgery (ANOVA *P *< 0.05). Different superscripted letters indicate significant pairwise differences between groups following significant time by surgery interaction (ANOVA *P *< 0.05). S = Sham, T = Transected.

There was a significant effect of surgery on the gene expression of GLUT4, whereas there was a significant effect of surgery, time and their interaction on GLUT4 protein expression (*P *< 0.05). Although not significant, GLUT1 mRNA was lower in both sham and transected muscle at 56 days post surgery. Pairwise comparison revealed that the GLUT4 protein expression was unchanged at 3 and 10 days in transected muscles but was significantly higher by 28 and 56 days post surgery (*P *< 0.05; Fig.6). However, gene expression levels for GLUT4 at all time points were significantly lower in the transected muscles (*P *< 0.05; Fig.6). GLUT1 gene expression was not affected by any factors, but GLUT1 protein expression was significantly affected by surgery (*P *< 0.05). GLUT1 protein expression was significantly greater posttransection surgery, although the effect was small (*P *< 0.05; Fig.6).

### Akt signaling

**Figure 7:**
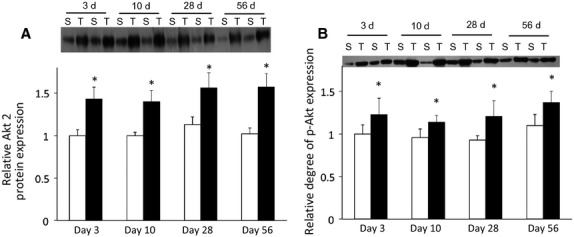
The response of Akt2 to chronic denervation suggests compensation to maintain insulin signaling in GC muscle. A. Total Akt2 expression is higher in denervated muscle. B. Amount of phospho-Akt is higher with denervation surgery. Mice were injected with either insulin (0.5 U/mouse) or saline 10 minutes prior to sacrifice. Only insulin injected animals are shown. Open bars represent sham surgery while closed bars represent denervation surgery. Expression presented relative to 3-day sham. Ponceau S staining was used to verify loading volumes for Western blots. Values are means ± SEM, (n = 7–9). *indicates a significant effect of surgery (ANOVA *P *< 0.05). Different superscripted letters indicate significant pairwise differences between groups following significant time by surgery interaction (ANOVA *P *< 0.05).

Surgery had a significant effect on protein expression of Akt2 (*P *< 0.05) with greater Akt2 protein expression post surgery (*P *< 0.05; Fig.7). Surgery also had the significant effect of higher Akt phosphorylation after transection (Fig.7). There was no difference in the expression of AS160 or p-AS160 at any of the time points (data not shown).

## Discussion

This is the first report of the response of the muscle glucose uptake system following long-term denervation. The effects of denervation on skeletal muscle are wide-ranging and include significant atrophy of muscle mass. In their review, Thomason and Booth (Talanian et al. 2010) noted that decreases in muscle mass are significant the first several weeks following surgery, but this rapid loss slows approximately 25–30 days post surgery. This is significant to our study as previous investigations that noted decrements in glucose uptake were limited to shorter time courses (Thomason et al. 1987; Block et al. 1991; Coderre et al. 1991; Henriksen et al. 1991; Lin et al. 2004). The observation that decreases in muscle mass were not noted after 25 days of disuse atrophy led to our hypothesis that the inhibition of glucose uptake seen in previous short-term denervation studies may not have been representative of the effects that chronic disuse atrophy has on glucose uptake. It has been suggested that changes in fiber type may increase glucose uptake. Previous research does not indicate changes in fiber type following prolonged transection in predominantly fast muscle (Booth 1977; Borisov et al. 2001). The EDL, TA, & GC are primarily “fast” muscles and so few changes in fiber type should be expected. Additionally, we saw no evidence of change to a more oxidative fiber-type as demonstrated by our metabolic gene expression data.

In our study, denervation of the sciatic nerve decreased muscle mass in soleus by 27% and 41% by 10 days and 56 days post surgery, respectively, values that are quite similar to those previously reported (Thomason and Booth 1990). Although greater in scope, reductions in mass by 10 and 56 days post surgery of tibialis anterior (40% and 59%) and gastrocnemius (50% and 68%) are also similar to those reported previously (Booth 1977). Furthermore, and in agreement with earlier reports (Booth 1977), we reported no differences in mass between 28 and 56-days post surgery. As there were no changes in mass for any of the sham surgery muscles over the course of the study, differences in mass could be accounted for as atrophy, not arrested development. The loss of mass was explained by reductions in mean myocellular CSA, which became significant 10 days post surgery. Similar reductions in mean CSA following denervation have been reported in skeletal muscle (Borisov et al. 2001; Wagatsuma et al. 2011) and diaphragm muscle (Aravamudan et al. 2006). Reductions in mean CSA were accompanied by increases in tissue fibrosis area, an effect of denervation or immobilization that has also been demonstrated previously (Pessin and Saltiel 2000; Wilkes and Bonen 2000; Arruda et al. 2007). Previous studies had focused on changes in glucose uptake shortly after surgery (3 h to 17 days post surgery), a period when apoptotic and necrotic pathways are most active. As the sciatic nerve is both motor and sensory, loss of sensory feedback may contribute to the observed phenotype. However, our data demonstrate that by 28 days post surgery, there was little evidence for continued atrophy demonstrated by the plateau in loss of muscle mass. Therefore, we investigated changes in glucose uptake to determine if there were indeed compensatory responses once apoptotic and necrotic activity had slowed significantly.

Following denervation surgery, decrements in insulin-stimulated glucose uptake have been noted as quickly as 3 h, although maximal effects were often not noted for 3 days (Thomason et al. 1987; Block et al. 1991; Coderre et al. 1991; Henriksen et al. 1991; Lin et al. 2004). Similarly, it has also been shown that between 3 days and 17 days post surgery, there was no change in the decrement in insulin-stimulated glucose uptake caused by denervation of the gastrocnemius muscle (Thomason and Booth 1990). Results from our functional glucose uptake assay show that insulin-stimulated glucose uptake was absent in the earliest time point (3 days post surgery). However, by 28 and 56 days post surgery, insulin-stimulated glucose uptake was greater in denervated muscles than in sham-surgery tissues. Likewise, basal glucose uptake was initially unaffected by denervation surgery, but was twofold higher in the 28 and 56 day groups. At this time point, appearance of immature, regenerating fibers may be occurring. Although we demonstrated no evidence for the presence or absence of immature fibers, it may be that the altered glucose uptake is due to their appearance. Regardless of the nature of the fibers, to determine the mechanisms responsible for the improved glucose uptake, we investigated expression of glucose transporters and components of the insulin-signaling pathway.

The expression of GLUT4 protein mirrored the changes in glucose uptake throughout our time course. The initially lower mRNA & protein expression (although not significant for protein) of GLUT4 at 3 days post transection were similar to those noted previously (Block et al. 1991; Coderre et al. 1991; Jensen et al. 2009). GLUT4 mRNA remained low throughout the experiment; however, GLUT4 protein expression in denervated muscles was significantly greater at the 28 and 56-day time points. These results are similar to those reported previously, which showed that in late-stage streptozotocin-induced diabetes, there are decreases in GLUT4 protein, but in the early stages GLUT4 protein levels are unchanged (Kahn et al. 1991). Therefore, it is possible that the diabetic phenotype was nascent in our model. Additionally, the decreases in GLUT4 mRNA without changes in GLUT4 protein expression suggest that the turnover of protein is slower than that of mRNA.

It has been shown previously that Akt plays a key role in glucose homeostasis; decreases in glucose uptake 24 h after denervation were attributed to decreases in Akt activation, as GLUT4 protein expression was unchanged (Henriksen et al. 1991; Wieman et al. 2007). Furthermore, overexpression of Akt in adipocytes from rats caused an increase in GLUT4 translocation, independent of insulin (Cong et al. 1997). In our study, both Akt2 and GLUT4 protein expression were increased in response to transection. Basal glucose uptake was also increased in the later time points in denervated muscles, although GLUT1 expression was unchanged. Via increased Akt activation, it has been shown in several cell lines that cytokines or other growth factors are able to increase basal glucose uptake by increased recycling of GLUT1 vesicles and promotion of cell surface integration without a change in GLUT1 protein levels (Williams and Goldspink 1984; Watson and Pessin 2001). The increased expression of both GLUT4 and Akt2 protein noted in the later time points, in concert with the increased insulin-stimulated glucose uptake seen at the later time points in our study further reinforces the idea that compensatory mechanisms involving the insulin signaling pathway act to restore the capacity for glucose uptake in response to long-term disuse atrophy. Although we did not measure the translocation of GLUT4, it is possible that some unknown, additional mechanism could have increased the localization of GLUT4 to the membrane, which could allow for higher glucose uptake.

The restoration of glucose uptake at later time points was cause for investigation into the effects that long-term denervation had on other metabolic pathways and on regulators of metabolism. The peroxisome proliferator-activated receptors (PPAR*α* and PPAR*δ*) and their co-activators (PGC-1*α* and PGC-1*β*) are known regulators of both lipid and glucose metabolism as well as mitochondrial biogenesis. PPAR*δ* has been shown to be strongly linked to glucose homeostasis; in human myotubes, PPAR*δ* agonists increased glucose metabolism (Koonen et al. 2004), whereas in *ob/ob* mice, pharmacological enhancement of PPAR*δ* activation improved glucose sensitivity and insulin response (Talanian et al. 2010). Additionally, mice lacking the PPAR*δ* gene were glucose intolerant (Kramer et al. 2006). PGC-1*β* has also been shown to affect glucose homeostasis, as transgenic mice overexpressing PGC-1*β* were lighter and had lower plasma insulin levels than pair matched controls when fed a high fat diet (Kahn et al. 1991). However, the effect that PGC-1*α* has on insulin resistance in obese mice is unclear as transgenic mice overexpressing PGC-1*α* were more insulin resistant and showed decreases in GLUT4 mRNA (Megeney et al. 1993), whereas PGC-1*α* null mice demonstrated improvements in glucose uptake and insulin response (Lees and Frank 2004).

The effects of long-term denervation on these metabolic regulators are less understood. Long-term denervation (42 days) in rats was shown to decrease PGC-1*α* expression by 70% (Adhihetty et al. 2007). In mice, PGC-1*α* and PGC-1*β* expression were decreased by 80% and 42%, respectively, 30 days after surgery (Turinsky 1987). In our study the expression levels for PPAR*α*, PPAR*δ*, PGC-1*α*, and PGC-1*β* were all decreased by ∼ 50% from day 10 through day 56. These data indicate that these regulators of metabolism are downregulated by denervation and do not appear to recover. There does not appear to be a direct connection between these metabolic regulators and the restoration of insulin sensitivity, although this appears to be in direct opposition to most studies, which show a strong correlation between metabolic regulation and glucose uptake. For example, it has been shown in muscle-specific knockout mice lacking the PGC-1*α* gene that caloric restriction was able to improve glucose homeostasis (Finley et al. 2012). Therefore, although there is a close relationship between these metabolic regulators and glucose homeostasis, other mechanisms (such as the increase in GLUT4 expression, and perhaps localization at the cell membrane, and Akt2 expression) appear able to compensate for the drop in expression with disuse atrophy.

The discrepancy between metabolic regulation and glucose uptake led us to investigate the effect that denervation would have on genes regulating enzymes involved in fatty acid transport and glycolysis. Due to the strong effect that PPAR*α*, PPAR*δ*, PGC-1*α*, and PGC-1*β* have on glucose and lipid metabolism and the observed reductions in their gene expression, it was not surprising that the expression profile for MCAD, M-CPT1, CD36, and CS followed similar patterns. Similar to glucose uptake, transport of fatty acids into skeletal muscle responds to changes in muscle contraction or insulin levels, and studies have shown an association between ineffective fatty acid transport and metabolic pathologies (Sigal et al. 2006; Jain et al. 2009; Glatz et al. 2010). Although the effects of long-term denervation on fatty acid transport are unknown, 7 days of disuse atrophy following denervation surgery (Klip and Pâquet 1990) increased palmitate uptake, although FAT/CD36 protein expression was unchanged. It was not surprising that the lower expression for these genes involved in fatty acid transport and glycolysis did not have an effect on glucose uptake.

To our knowledge, this is the first report of the effects that long-term denervation has on functional glucose uptake. We demonstrated that both basal and insulin-stimulated glucose uptake in transected muscles were greater than control at 56 days post surgery, whereas the expression of metabolic genes in transected muscles was not restored. Although the exact mechanisms for the improvement in glucose uptake remain unclear at this time, it appears that the greater protein expression we noted for both GLUT4 and Akt2 shown in transected muscles are partially responsible. Although GLUT4 protein was increased, it is possible that other mechanisms, such as increased translocalization of GLUT4 to the plasma membrane, increased fusion with the plasma membrane, or functional activity of GLUT4 are responsible for the increase in glucose uptake. Further inquiries into the mechanisms underlying this response are clearly warranted.

## Supporting Information

**Table S1.** Primer sequences for qPCR.
