# Supplementary material for: Compensatory responses of the insulin signaling pathway restore muscle glucose uptake following long-term denervation
Source: Physiol Rep. 2015 Apr 20;3(4):e12359. doi: 10.14814/phy2.12359 (PMC4425965; doi:10.14814/phy2.12359)
Supplement: Supplementary file 1 — Table S1. Primer sequences for qPCR. [file phy20003-e12359-sd1.docx]

Supplemental Table 1. Primer sequences for qPCR.

| **Gene** | **Forward** | **Reverse** |
| --- | --- | --- |
| PPARα | ACTACGGAGTTCACGCATGTG | TTGTCGTCAACCAGCTTCAGC |
| PPARδ | TCACCGGCAAGTCCAGCC | ACACCAGGCCCTTCTCTGGCT |
| PGC-1α | CGGAAATCATATCCAACCAG | TGAGGACCACTAGCAAGTTTG |
| PGC1β | TCCAGAAGTCAGCGGCCT | CTGAGCCCGCAGTGTGG |
| MCAD | GGCTCCTGAGAAGTGTTTCTC | GGCTCCTGGTTGTGAGC |
| MCPT1 | TCTAGGGAATGCCGTTCAC | GAGCACATGGGCACCATAC |
| CD36 | CAAGCTCCTTGGCATGGTAGA | TGGATTTGCAAGCACAATATGAA |
| PFK | CGTTGAGGTAGGAATACTTCTGCA | ACCTCTTCCGAAAGGAGTGGA |
| CS | CAAGCAACATGGGAAGA | GTCAGGAAGAACCGAAGTCT |
| GLUT4 | ATCATCCGGAACCTGGAGG | GTCAGACACATCAGCCGAGC |
| GLUT1 | TCGTTGGCATCCTTATTGC | ACGAAGACGACACTGAGCAG |
| 36B4 | AGATGCAGCAGATCCGCAT | ATATGAGGCAGCAGTTTCTCCAG |
